# Fabrication of Polysaccharide-Based Halochromic Nanofibers via Needle-Less Electrospinning and Their Characterization: A Study of the Leaching Effect

**DOI:** 10.3390/polym14194239

**Published:** 2022-10-10

**Authors:** Beste Elveren, Silvo Hribernik, Manja Kurečič

**Affiliations:** 1 Laboratory for Characterization and Processing of Polymers, Faculty of Mechanical Engineering, University of Maribor, Smetanova Ulica 17, 2000 Maribor, Slovenia; 2 Institute of Automation, Faculty of Electrical Engineering and Computer Science, University of Maribor, Koroska cesta 46, 2000 Maribor, Slovenia

**Keywords:** halochromism, responsive polymers, polysaccharides, electrospinning

## Abstract

Responsive materials, i.e., smart materials, have the ability to change their physical or chemical properties upon certain external signals. The development of nanofibrous halochromic materials, specifically combining the pH-sensitive functionality and unique nanofiber properties, could yield interesting new applications, especially when the common problem of dye leaching is successfully tackled. Therefore, in this article, we studied the fabrication process of polysaccharide-based halochromic nanofibrous materials by using a combination of various halochromic dyes (bromothymol blue, bromocresol green, and thymol blue) and cellulose acetate in a spinning solution using a one-pot strategy. The inhibition of leaching was addressed by using a complexing agent: poly-diallyl-dimethylammonium chloride (PDADMAC). The preparation of hybrid spinning solutions, their characterization, and ability to form continuous nanofibers were studied using a high production needle-less electrospinning system. The produced hybrid solutions and nanofibers were characterized, in terms of their rheological properties, chemical structure, morphology, and functionality. Fabricated nanofibrous halochromic structures show a clear color change upon exposure to different pH values, as well as the reduced leaching of dyes, upon the addition of a complexing agent. The leaching decreased by 61% in the case of bromocresol green, while, in the case of bromothymol blue and thymol blue, the leaching was reduced by 95 and 99%, respectively.

## 1. Introduction

In the last decade, responsive smart materials became intriguing because of the dynamic and mostly reversible changes they introduce as a result of an external stimuli. In this area of material research, electrospun nanofibers, with their unique characteristics, such as an extremely high specific surface area, high porosity, small pore size, and high absorbance capacity, occupy a prominent position [1]. Nanofibrous structures, designed by randomly deposited small diameter sized nanofibers (diameters from ten to few hundred nanometers), facilitate liquid transport in the material, due to the large and easily accessible pores among fibers, thus leading to fast response to chemical stimuli and making them promising materials for several fields, along with sensing applications [2,3]. Electrospinning (ES) can be used to produce various functional fibers with its advantages of low cost, easy tuning of morphology, and capacity to produce continuous long fibers [4]. The conventional and frequently studied approach to develop electrospun nanofibers often uses a needle-like nozzle (needle electrospinning). Ghazalian et al. produced chitosan-polycaprolactone core-shell nanofibers using co-axial needle electrospinning with tetracycline hydrochloride encapsulated as an antibacterial source [5]. Although the study was successful and shows great promise in biomedicine, up-scaled production of the antibacterial nanofibers remains a challenge. Despite the enormous application potential, needle electrospun nanofibers meet difficulties in broad applications in practice, due to the lack of an economic and efficient way to up scale the electrospinning process [6]. Recently, needle-less electrospinning has emerged with the ability to produce nanofibers on large scales [7]. Needle-less electrospinning features nanofiber creation directly from an open liquid surface, where numerous jets are formed simultaneously, without the influence of capillary effect that is normally associated with needle-like nozzles [8]. In a study by Karim et al., needle-less electrospinning was used to produce antibacterial and antioxidant packaging material to improve the shelf-life of perishable food [9]. Zein nanofibers containing tetradecane and cinnamaldehyde were investigated, and they were able to improve the shelf-life, when compared to the control group. Yarin and Zussman reported a needle-less electrospinning system that used a magnetic field to initiate the jet formation for the development of halochromic nanofibers. They compared the production rate of polyethylene oxide (PEO) nanofibers using multi-needle electrospinning and needle-less electrospinning, resulting in an increase of productivity by 12 times using needle-less electrospinning [10]. In another study, Panda et al. optimized the diameter size of Nylon 6, produced through a needle-less electrospinning process by administering the central composite design (CCD) and response surface methodology (RSM) models in their experimental process [11]. These models provide high-quality prediction of the experimental output, as well as efficient study of the multivariable systems, while predicting the optimal conditions for the experiments.

Smart materials often contain halochromic dyes that consist of a pH-sensitive chromophore, responding to environmental pH by a color change [12]. Depending on the molecular structure of the chromophore, the specific coloration effects can be observed at defined ranges of pH values. The incorporation of a halochromic dye into a desired nano-fibrous matrix could, therefore, be exploited to achieve a visual sensor [13], with the ability to detect the effects by naked eye. The pH sensitive electrospun nanofibers can find wide applications in the areas of the smart packaging of food and beverages [14], textiles [15], tissue engineering [15,16,17,18], water filtration, analysis of microenvironmental pH change involved in various biological processes [19], wound dressings, and microbiological studies [20]. Indeed, the nanofibrous matrix represents an ideal carrier for halochromic dyes, aiming to achieve long-lasting and fast responsivity.

Schueren et al. used co-spinning to develop pH-sensitive polyamide 6.6 nanofibers for a limited range of pH values [15]. The study indicates that dye–polymer interactions depend on relatively weaker interactions, such as van der Waals and hydrogen bonding. Moreover, the halochromic sensor was affected by poorly soluble dye components. Agarwal et al. produced nylon electrospun nanofibers containing five different pH indicators in specific proportions to cover a wide range of pH values [13]. These smart electrospun nanofibers detected changes in the solution’s pH (from pH 1 to 10), based on the characteristic colors for each pH value. As a result, the study achieved halochromic responsiveness by the addition of multiple pH indicators, without compromising nanofiber integrity. Van der Schueren et al. explored the feasibility of using commercial pH-indicator dyes for dye-doping polyamide 6.6 (PA66) nanofibers, which resulted in a wide range of nanofibrous membranes showing halochromic behavior, which could differ from the indicator dyes in solution [15]. The pH-range of the color change shifts and/or broadens; even the color itself can be influenced, all depending on the strength and mode of interaction between the polymer matrix and dye. Following two studies incorporating the commercial azo indicator dye Nitrazine yellow (NY) in polyamide (PA) or polycaprolactone (PCL) and PCL/chitosan nanofibers, they studied the influence of a polymer nanofibrous matrix on the halochromic behavior of an indicator dye in more detail [21,22]. Based on these studies, it can be concluded that the changing microenvironment of an indicator dye, due to incorporation into nanofibers, influences the halochromic behavior on three levels: the (i) absorbance spectrum (i.e., the colors), (ii) dynamic pH-range (pKa, broadening), and (iii) response time. For instance, NY-doping of PA6 and PA66 nanofibers resulted in a slight shift of the acidic and alkaline wavelength maxima, as well as a shift and broadening of the dynamic pH-range. When using PCL as the matrix polymer, the halochromic behavior of NY is even completely suppressed. The addition of chitosan to this system restores the halochromic behavior again. Moreover, the PCL/chitosan nanofibers have superior wetting behavior, in comparison to the PCL nanofibers, effectively lowering the response time from 3 h to 5 min. Thus, these studies show that a well-considered choice of nano-fibrous matrix is crucial. 

Recently, there are some reports on incorporating halochromic dyes into polysaccharide-based matrix (which have represented a viable alternative to synthetic polymers), in order to improve the biological use of these smart sensor systems. In previous research, Kurečič et al. investigated a pH-responsive drug release mechanism for wound dressing applications combined with the pH-responsive dye [23]. The study focused on the integration of bromocresol green (BCG) and benzocaine in a cellulose acetate (CA) matrix and observed the drug release and color change. Another study focused on using chitosan and poly (acrylic acid) to induce a pH response, in terms of the swelling ratio [24]. Cheng et al. reported that chitosan/polyethylene oxide composites can be used for controlled drug release in wound dressing applications [25]. While tackling the biodegradation and biocompatibility of halochromic smart nanofibrous materials by using naturally derived polymers, the main problem of dye leaching is still present. Leaching of the dye has been one of the major problems when producing halochromic materials, since most of the methods depend on weaker molecular interactions. This drawback can be eliminated, to a certain degree, when the dye is crosslinked into polymeric structure or a chemical bond between the dye and polymer is achieved. Schueren et al. studied the bonding between the halochromic dye methyl red (MR) and cellulose-based textile fabrics (scoured and bleached cotton) via the use of a silane source glycidoxypropyltrimethoxysilane (GPTMS) [18]. In another study by Rosace et al., a chemical bond is established between the halochromic dye resorufin (RF) and GPTMS via sol-gel method [26]. However, in order to achieve this goal, a corrosive and hazardous catalyst, 1-methylimidazole, had to be used, which limits the applications of this technique. Additionally, some reports can be found on the prevention of leaching by using a complexing agent and trapping the dye molecule to the polymeric structure. Meyer et al. reported that the leaching of sulfonphthaleine-based dyes decreases subsequently in the presence of poly-diallyl-dimethylammonium chloride (PDADMAC) [27]. Nanofibrous polyamide 6 (PA6) non-vowens were integrated with several sulfonphthaleine-based halochromic dyes and investigated for their leaching mechanisms. PDADMAC is a complexing agent that has been used to suppress the leaching of the dye from the polymeric matrices, mostly used in textile industry [28]. It prevents the migration of dye molecule from the matrix by the ionic interactions [29].

**Figure 1:**
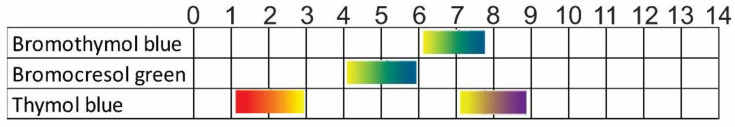
pH ranges of the halochromic dyes.

In this study, the development of pH-responsive polysaccharide-based hybrid nanofibers with reduced dye leaching is presented via the addition of the complexing agent, PDADMAC. Halochromic dyes were homogeneously integrated into the spinning solutions of different CA concentrations, i.e., anionic dyes: bromocresol green (BCG), bromothymol blue (BTB), and thymol blue (TB), to provide the required responsivity to pH change. Figure 1 presents the color change of the halochromic dyes used in this study, covering almost the whole pH-range. A series of halochromic sulfonphthaleine dyes were chosen for this study, with the aim of achieving a greener approach towards the production of polysaccharide-based halochromic smart nanofibers. BCG is often used as a model halochromic dye in bio applications because of its responsivity range and reactivity. BTB was chosen, due to its similar anionic structure to BCG, but less bromine (Br) content, in order to reduce the toxicity. Finally, to achieve our aim of greener approach, thymol blue (TB) was included in this study. Since Br is considered toxic, even in trace amounts, BCG and BTB would have limited applications, especially in biomedicine. Even though there are several reports using BCG as a sensor, when in contact with biological system, leaching of the dye can cause severe effects, due to Br within its structure. Therefore, TB, as an alternative dye, can have the desired impact towards achieving our goal. The produced hybrid solutions were characterized, in terms of their rheological properties and conductivity. After the hybrid solutions were characterized, they were electrospun, and the resulting nanofibers were evaluated according to their morphology, structure, responsiveness, and suppressed dye leaching ability.

## 2. Materials and Methods

Cellulose acetate (CA, Mn = 30.000 by GCP, acetyl content: 39.8 wt%), acetic acid (AcOH ≥ 99.8%), bromocresol green (BCG), bromothymol blue (BTB), thymol blue (TB), and poly-diallyldimethylammonium chloride (PDADMAC, Mw: 200.000–350.000) were all purchased from Sigma Aldrich, Germany. For all experiments, ultra-pure water was used, prepared from a Millipore water purification system (Merck KGaA, Darmstadt, DE; resistivity = 18.2 MX cm). All buffer solutions (pH = 4,7,10) were purchased from Reagecon Diagnostics Ltd., Ireland.

### 2.1. Hybrid Solution Preparation

Homogeneous CA solutions were prepared at four different concentrations (9, 11, 13, and 15 wt%) by dissolving appropriate amounts of CA in 85% AcOH and mixing for 24 h. A total of 4 wt% of complexing agent (PDADMAC) and 0.3 wt% of halochromic dye were added to individual CA solutions to obtain hybrid spinning solutions by mixing until homogeneous mixtures were reached (abbreviated as R-P-X-Y). Different halochromic dyes (BCG, BTB, and TB) were utilized to obtain a range of pH values. The mixing of the hybrid solutions was achieved by using a mechanical mixer (IKA Eurostar 20 digital, DE) at 300 rpm for 24 h. All the hybrid spinning solutions were characterized, regarding their viscosity, conductivity, and color changing properties. Reference hybrid spinning solutions without the complexing agent PDADMAC were also prepared, in order to compare the effect of complexing agent on spinnability and leaching properties (abbreviated as R-X-Y). To do so, CA was prepared at different concentration from 9 to 15 wt% in 85% AcOH by mixing for 24 h using a mechanical mixer (IKA Eurostar 20 digital, DE) at 300 rpm, and 0.3 wt% halochromic dye was added to individual CA solutions.

All the abbreviations for prepared hybrid solutions are provided in Table 1. Reference hybrid solutions were named R-X-Y, and hybrid solutions were named H-P-X-Y, where P represents the presence of PDADMAC, X represents the dyes, and Y represents the concentration of CA.

### 2.2. Rheology and Conductivity Measurements of Spinning Solutions

The rheology of reference and hybrid spinning solutions was investigated by using Anton Paar GmbH, Rheometer MCR302 (AT), with cylindrical measuring system from 0.001 to 1000 1/s shear rate at room temperature. The conductivity of the hybrid solutions was measured using a Mettler Toledo International Inc. conductometer (CH), accessorized with conductivity probe InLab 710 for highly acidic conditions.

### 2.3. Electrospinning

Electrospinning was performed using a pilot scale needle-less electrospinning apparatus (NanoSpider NS LAB 500, ElMarco s.r.o, Liberec, CZ), which allows the formation of nanofibers on a support material with 50 cm width and infinite length (i.e., a continuous production process). In the electrospinning process, wired electrodes were used as both upper and lower electrode, and different process parameters were varied to optimize the electrospinning conditions. The applied voltage was altered from 50 to 75 kV in 5 kV increments. Electrode distance was also adjusted from 100 to 190 mm in 10 mm increments. For optimal electrospinning of the different hybrid solutions, 140 mm electrode distance and 65 kV voltage were chosen for the comparison between different concentrations of CA and addition of different dyes. The ambient temperature was 19.2 ± 0.9 °C, and the ambient humidity was 27 ± 2%. The electrospinning duration was optimized at 30 min to obtain adequate samples. Hybrid solutions were electrospun on aluminum foil, as a support material, with dimensions of 300 × 400 mm. 

All the abbreviations for produced nanofibers are provided in Table 2. Nanofibers produced from their respective hybrid solutions were named N-P-X-Y, and reference nanofibers produced from their respective hybrid solutions were named RN-X-Y, where P represents the presence of PDADMAC, X represents the dyes, and Y represents the concentration of CA.

### 2.4. Morphology Analysis

The morphology of the electrospun nanofibers was observed using a scanning electron microscope (Carl Zeiss FE-SEM SUPRA 35 VP, Zeiss, Oberkochen, Germany) at an accelerating voltage of 1 kV after sputter coating the samples with a thin layer of palladium using a Benchtop Turbo sputtering device (Denton Vacuum LLC, Moorestown, NJ, USA). The diameter of the nanofibers was measured using Image J (1.53a, National Institute of Health, Stapleton, NY, USA) software. 

### 2.5. Characterizations of the Dyes

In order to evaluate the interactions between the dyes and PDADMAC, pH-potentiometric titration in water was performed, in order to quantify the charge present in the solutions for pure dye and PDADMAC–dye mixtures. For potentiometric titrations, 10 mg of dyes were titrated individually, starting from acidic to alkaline, with 0.1 mol/L HCl and 0.1 mol/L KOH as titrants. Additionally, mixtures of 4 wt% PDADMAC (1.2 g) and 0.3 wt% (0.09 g) of each dye were individually prepared and titrated, following above procedure. A two-burette auto-titration unit T70 (Mettler Toledo International Inc., Greifensee, Switzerland), and a glass pH electrode DG-111 SC (Mettler Toledo International Inc., Greifensee, Switzerland) was used to measure the pH of the solution continuously. Purging with nitrogen gas ensured an inert atmosphere during the measurements. The ionic strength was set to 0.1 mol/L (adjusted by the addition of 3 mol/L KCl). The pKa values of each dye and mixtures were calculated and presented. Data analysis and calculations were performed according to procedures described elsewhere [30]. Additionally, the dyes were dissolved in 1× PBS solution individually to obtain a concentration of 1 × 10^−5^ M. UV/VIS Spectrophotometer Agilent Cary 60 (Agilent Technologies Inc., Santa Clara, CA, USA) was used to scan the prepared solutions, in order to determine the unique wavelength maxima (λ_max_) values needed for further analysis regarding responsivity and leaching. 

### 2.6. Responsivity Measurements

Monitoring of the responsivity of the electrospun nanofibers was performed with several buffer solutions with different pH values. Considering the color change of the halochromic dyes, which was shown in Figure 1, pH values of 4, 7, and 10 were chosen. Nanofiber mats were cut into 40 × 40 mm squares to optimize the measurement. The nanofibers were photographed before the contact with individual buffer solution. Afterwards, samples were dipped into the respective buffer solutions for 15 s and photographed after 15 s of drying. This procedure was repeated 4 times for each nanofiber mat at selected pH values. For photographing the samples, a commercial 300 × 300 × 300 mm cubic Photo LED box was used with 1200 lumen power LED’s. White light at 6500 °K was used to obtain standardized illumination on the samples. A mobile phone that has quadruple camera with 64-megapixel wide, 12-megapixel ultrawide, 5-megapixel macro, and 5-megapixel depth lenses was used to capture the images from 300 mm height, with no magnifications, on a white surface. 

The color of samples was evaluated, in terms of the CIE L*a*b* color system, where L*, a*, and b* were the coordinates of the color in the mathematical combination of a Cartesian and cylindrical coordinate system, based on the theory that color is perceived as L* (lightness, from 0 for absolute black to 100 for a perfect white), a* (green—negative axis and red—positive axis), and b* (blue—negative axis and yellow—positive axis). The measurements were performed within a spectral range of 400–700 nm wavelengths using a two-ray spectrophotometer Spectraflash SF600 Plus (Datacolor, Trenton, NJ, USA) at standard illuminant D65 (LAV/Spec. Incl., d/8, D65/10°), from which the CIE color values were calculated using the Datacolor Match Textile 2.6.3.19R software (Datacolor, Trenton, NJ, USA) [31,32]. A Xenon halogen lamp was used as the light source.

### 2.7. Analysis of Dye Leaching

Extent of dye leaching from each nanofibrous mat sample was analyzed using UV/VIS Spectrophotometer Agilent Cary 60 (Agilent Technologies Inc., Santa Clara, CA, USA). Specified amounts of nanofibers (0.003 g) was immersed in separate 1× PBS solutions (5 mL), and leaching of the dyes was investigated by monitoring increase in absorbance at maxima wavelengths, characteristic for each respective dye. A single read UV/VIS measurement was performed at the beginning and end of 24 h immersion. 

## 3. Results and Discussion

### 3.1. Optimization of Hybrid Solutions

**Figure 2:**
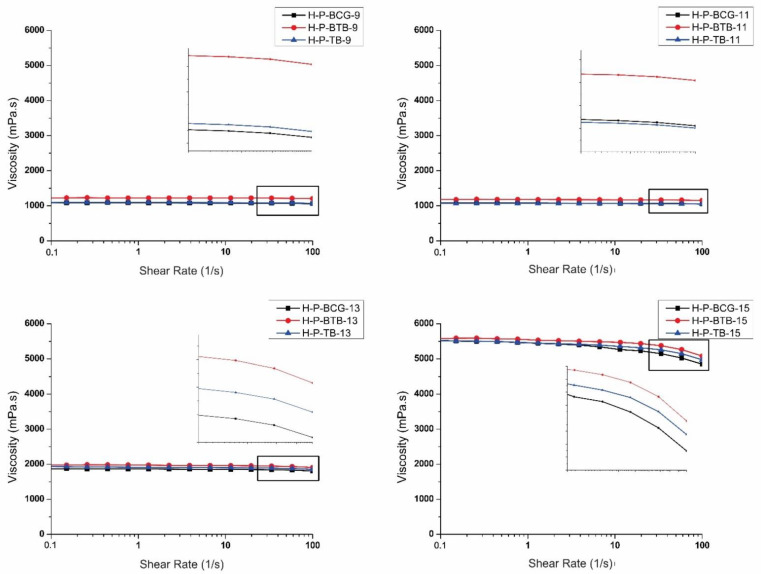
Viscosity curves of hybrid solutions with different concentrations of CA.

**Figure 3:**
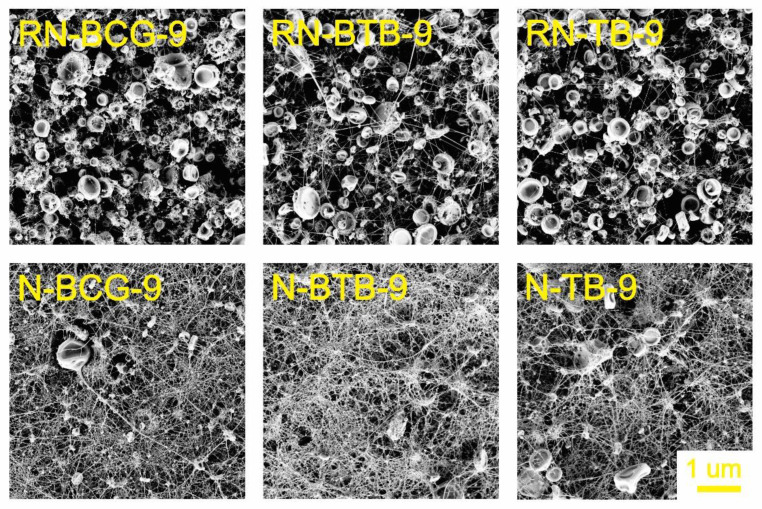
SEM images of electrospun nanofibers from 9 wt% CA containing hybrid solutions, compared with their respective reference nanofibers (for all samples, 5K × magnification images are presented).

For the successful fabrication of nanofibrous materials using the electrospinning process, it is essential to evaluate and optimize the spinning solution parameters according to their viscosity and conductivity. With optimal spinning solution parameters in place, formation of smooth, uniform, and homogeneous nanofibers can be ensured [33]. There are several reports dealing with the importance and influence of these parameters on nanofiber formation using the needle electrospinning process [34]. The optimization of these parameters is even more pronounced in the needle-less electrospinning [33,35], where the spontaneous formation of Taylor cones on a free liquid surface, ejection of polymer jets, and consequent nanofibers formation is solely dependent on the solution, as well as the ambient and process parameters [36,37]. Therefore, the viscosity and conductivity of the prepared hybrid solutions were characterized, and the results are shown in Figure 2 and Figure 3, respectively. 

In Figure 2, the expected increase in the viscosity of the prepared spinning solutions corresponding with the increase of CA concentration is shown; consequently, an increase of polymer chain entanglements in the solution is also proposed [23,38,39]. In Figure 2, one can observe the spinning solutions, especially with lower concentrations of 9 and 11% CA, as Newtonian liquids; however, upon closer inspection of the behavior at shear rates from 30 to 100 1/s [40], a slight shear thinning behavior was observed. By increasing the concentration of CA, the shear thinning behavior was more pronounced, and the samples H-P-BCG-15, H-P-BTB-15 and H-P-TB-15, which have the highest concentration of CA, showed shear thinning, i.e., a decrease in viscosity, already at the shear rate of 30 1/s. Generally, this behavior is explained by the alignment of the polymeric chains, with increasing shear strain [41]. Moreover, this behavior consequently contributes to the electrospinning process. In a study by He et al., they investigated the shear strain effect by using different types of needles for electrospinning and concluded that the spinning solution’s shear rate was highly interconnected with the nanofiber production [42]. This can be related to the extent of polymer chain entanglements, due to the increased number of polymer molecules in the solutions, forming the stabile and continuous polymer jets [39]. The viscosities of the reference hybrid solutions with different concentrations of CA, namely R-BCG-9, R-BCG-11, R-BCG-13, and R-BCG-15, were determined as 996.1, 976.3, 966.6, and 4721.7 mPa.s, respectively. The viscosities of pure CA, without the dye and PDADMAC, were previously reported by Kurečič et al. [23], where they prepared pure CA nanofibers using the same protocol. The reported values of pure CA solutions at 12, 15, and 17% were 958, 4755, and 10,143 mPa.s, respectively, indicating that no interaction between the CA and dyes took place in the reference hybrid solutions. With the addition of PDADMAC, we observed an increase in the solution’s viscosity, in the case of BCG, namely the samples H-P-BCG-9, H-P-BCG-11, H-P-BCG 13, and H-P-BCG-15 (1090.1, 1076.3, 1866.6, and 5611.7 mPa.s, respectively), which can be attributed to the ionic interactions between PDADMAC, a charged electrolyte, and the BCG dye, which contains four Br-ions in its structure. Bernardino et al. and Ozaki et al. concluded that the contribution of the direct effect of ionic interaction is predominant [43,44]. The same trend can also be observed for BTB containing samples H-P-BTB-9, H-P-BTB-11, H-P-BTB-13, and H-P-BTB-15 (1221.3, 1206.4, 1972.6, and 5549.5 mPa.s, respectively). Lastly, TB containing samples, namely H-P-TB-9, H-P-TB-11, H-P-TB-13, and H-P-TB-15 (1088.7, 1075.3, 1912.7, and 5536.4 mPa.s), follow the behavior described above.

In the case of measured conductivity values, presented in Table 3, the samples H-P-BCG-15, H-P-BCG-13, H-P-BCG-11, and H-P-BCG-9 (400.4, 448.7, 598.8, and 342.3 µS.cm, respectively) show that the addition of PDADMAC results in a significant increase (up to 10-fold) in conductivity, in comparison to the reference hybrid solutions R-BCG-15, R-BCG-13, R-BCG-11, and R-BCG-9 (73.16, 114.7, 268.6, and 210.9 µS.cm, respectively). These results also correlate with our previous study (Kurečič et al.), reporting on pure CA solutions, indicating that the increase of conductivity is solely due to the addition of PDADMAC [23]. Hayati et al. have shown that highly conductive solutions are extremely unstable in the presence of a strong electric field, which results in a dramatic bending instability, as well as a broad diameter distribution [45]. Generally, electrospun nanofibers with the smallest fiber diameter can be obtained with the highest electrical conductivity, with decrease in the size of the fibers, resulting from the increased electrical conductivity [46,47,48,49,50]. Even though the highest conductivity was achieved with the samples H-P-BCG-13, H-P-BTB-13, and H-P-TB-13 (Table 3), SEM images show that homogeneous fiber formation occurs with the samples H-P-BCG-15, H-P-BTB-15, and H-P-TB-15. Therefore, it can be concluded that conductivity, as well as viscosity, plays an important role for the needle and/or needle-less electrospinning process and should be considered to be in synergy [33]. This was also proven by Bai et al., who have shown that the most conductive solutions do not necessarily result in the optimal formation of nanofibers [51].

### 3.2. Electrospinning of Hybrid Solutions and Characterization of Nanofibers

**Figure 4:**
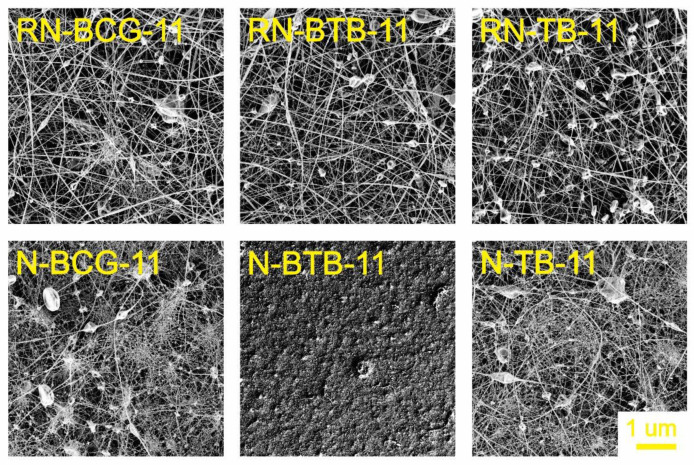
SEM images of electrospun nanofibers from 11 wt% CA containing hybrid solutions, compared with their respective reference nanofibers (for all samples, 5K × magnification images are presented).

**Figure 5:**
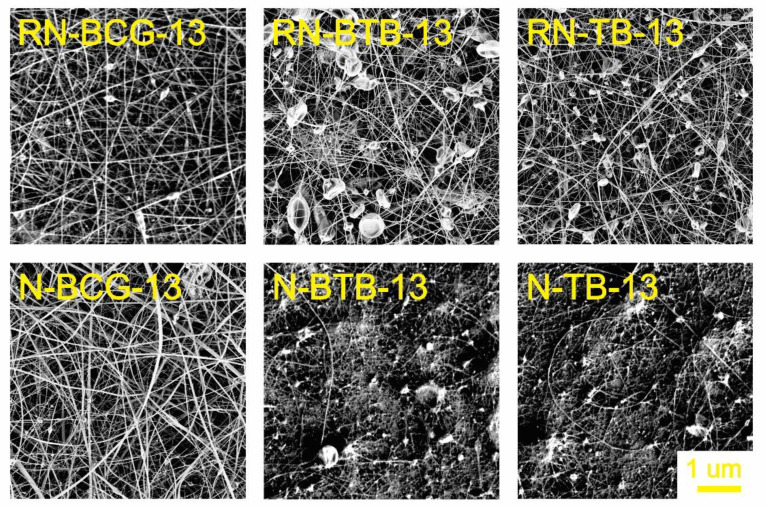
SEM images of electrospun nanofibers from 13 wt% CA containing hybrid solutions, compared with their respective reference nanofibers (for all samples, 5K × magnification images are presented).

**Figure 6:**
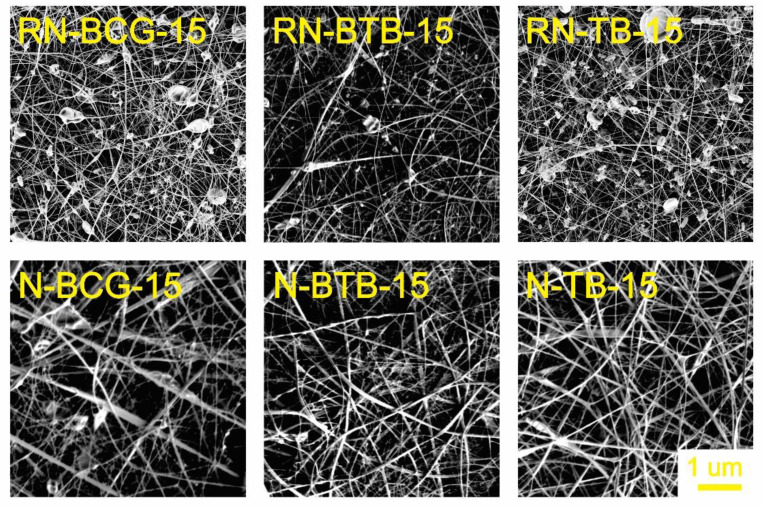
SEM images of electrospun nanofibers from 15 wt% CA containing hybrid solutions, compared with their respective reference nanofibers (for all samples, 5K × magnification images are presented).

Morphology is an important characteristic of nanofibers, closely connected with their function, which is, in turn, connected to the active surface area. The morphology can be affected by the solution’s properties, as well as the process and ambient conditions [52,53,54]. During our study, it was observed that the viscosity and conductivity of the hybrid solutions are the most influential parameters in the process of nanofiber formation. As seen from Figure 3, Figure 4, Figure 5 and Figure 6, there is a significant difference in the fibers’ morphologies, due to the concentration of CA and addition of PDADMAC. With an increasing CA concentration, more uniform and smooth nanofibers are formed. At low concentrations of CA, small particles shaped as concaved microdiscs are observed. In Figure 3, reference nanofibers without PDADMAC at the lowest CA concentration (RN-X-9) show an abundance of concaved microdiscs (average microdisc diameter = 2.76 ± 0.66 µm), while hybrid fibers, with the addition of PDADMAC (N-P-X-9), exhibit very thin nanofibers (average nanofiber diameter = 16.09 ± 0.06 nm) along with small proportion of different sized microdiscs. With the increasing CA concentration, microdisc formation is reduced, and nanofiber formation is in favor for RN-X-11 and N-P-X-11 samples, which can be seen in Figure 4. As mentioned above, hybrid solutions with 13 wt% CA, namely R-X-13 and H-P-X-13, possess the highest values of conductivity; therefore, it was expected that these hybrid solutions would produce uniform nanofibers. However, as already explained, due to different interactions between the components, these samples still have the presence of microdisc-like particles (Figure 5). A low solution viscosity causes the polymer jets in the electrospinning tub to disrupt into droplets and compounds the effect of surface tension (electrospraying). This phenomenon is also described in a previous study by Roemhild et al. for needle-less electrospinning [38]. The formation of the disc-shaped particles (microdiscs) occurs during the Taylor cone formation by evaporation of solvent. After the polymer droplet is ejected from the electrode, it is disrupted by evaporation of the trapped solvent on the pathway to the collecting electrode, which causes the formation of the biconcave-shaped microdiscs. By increasing the CA concentration to 15%, more uniform and smooth nanofibers are produced, due to the significant increase in solution’s viscosity (Figure 6).

The addition of PDADMAC to the reference hybrid solutions increased the conductivity of the samples and improved the electrospinning ability. As mentioned before, highly conductive electrospinning solutions produce nanofibers with smaller diameter size, along with the production of disc-like particles. On the contrary, the low conductivity of the reference hybrid solutions introduced a challenge for the electrospinning process. The effect of the viscosity and conductivity of the reference hybrid solutions results in small particle formation, along with challenges in electrospinning, as well as obtaining a significant amount of the sample [55,56,57]. Figure 6 shows that the H-P-BCG-15, H-P-BTB-15, and H-P-TB-15 solutions produce uniform nanofibers without unwanted inclusions. Therefore, for the investigation of dye leaching, the 15 wt% CA concentration was chosen for the further characterizations.

**Figure 7:**
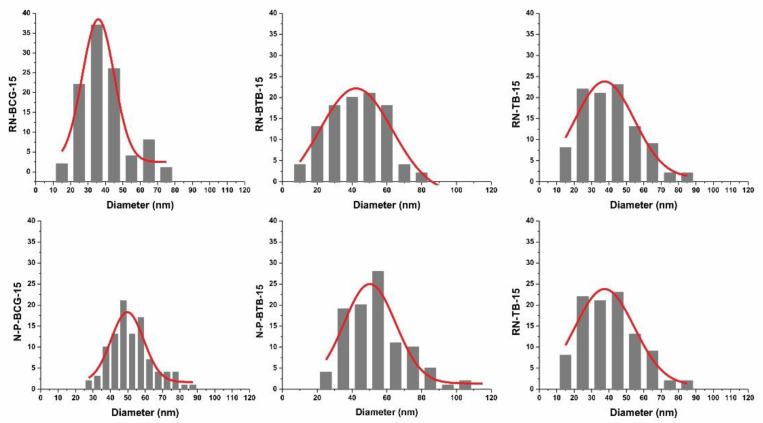
Size distributions of the reference and hybrid nanofibers from the samples containing 15 wt% CA.

Figure 7 shows the comparison of the nanofibers’ diameters from the reference and hybrid nanofibers, with 15% CA. Reference nanofibers without PDADMAC, namely RN-BCG-15, RN-BTB-15, and RN-TB-15, show 35.91 nm ± 1.20, 42.29 nm ± 1.63, and 37.53 nm ± 1.87 diameter sizes, respectively. In combination with Figure 6, we can also see that the addition of the dye has no influence on the production of nanofibers and their morphology and size, which can also be expected, according to the spinning solutions’ viscosity and conductivity measurements. On the other hand, hybrid nanofibers with the addition of PDADMAC, N-P-BCG-15, N-P-BTB-15, and N-P-TB-15 show slight increase in diameter size, 49.76 nm ± 1.12, 50.21 nm ± 2.14, and 49.69 nm ± 0.92, respectively. In addition, the SEM images (Figure 6) of nanofibers with BCG and BTB dye (N-P-BCG-15 and N-P-BTB-15) showed some individual microdisc formation, while, in the case of TB (N-P-TB-15) dye, this was not observable. This formation can be caused by the fact that the N-P-BCG-15 and N-P-BTB-15 show higher conductivity, compared to N-P-TB-15 sample, and an indication of the electrospraying phenomena. However, it should be noted that, even though high conductivity of the spinning solutions is desirable, as described before, highly conductive solutions can also result in electrospraying. This description completely depends on the characteristics of the spinning solutions.

### 3.3. Characterization of the Dyes

To investigate the possible complexation between the components in hybrid solutions and nanofibers, halochromic dyes were titrated individually and in combination with the PDADMAC, in order to evaluate the pKa values. The acid dissociation constant (pKa) can be defined as the strength of an acid within different solutions [58]. It can be measured or calculated from the half equivalence point through a titration process. The half-equivalence point on the curves indicates several characteristics. Firstly, it can be defined as the point at which half of the original analyte has reacted with the titrant. Secondly, it is also the point at half the volume of the equivalence point. Lastly, at this point, the concentration of the acid is equal to the concentration of its conjugate base [59]. According to the data obtained, the pKa values of the dyes were identified as 4.9 and 7.27 for BCG and BTB, respectively. Theoretically, BCG and BTB have 4.9 and 7 as pKa values [27]. Uniquely, TB has 2 pKa values, and they are 2.48 and 8.10, experimentally. In the literature, the pKa values for TB were identified as 1.65 and 8.9, and they are in correlation with the values obtained from the experiments [27].

When titrated as mixtures (PDADMAC and dye), the pKa values of the mixtures change and shift towards higher values. It can be clearly seen that the charges present in pure dye and the mixtures have very different characteristics. During the titration process, along with pH increase, the negatively charged particles drastically increase for the prepared mixtures. It can also be concluded that, for the dye alone, it is a slow change, and the amount of negatively charged particles are low.

From the calculations, it was found that the mixture of BCG and PDADMAC had 5.12 pKa, which indicates that an interaction between PDADMAC and BCG dye [60]. The shift in pKa indicated that PDADMAC and BCG dye form a complex in a way that deprotonation of the newly formed structure requires a slightly more basic environment. Additionally, the same trend is observed for the PDADMAC and BTB mixture. The shift is from pKa 7.27 to 8.09. Therefore, the same conclusion, i.e., that PDADMAC and BTB interact with each other, can be reached. Regarding the mixture of PDADMAC and TB, the calculations indicate that the new pKa value lies between pH 5 and 6. Since the first pKa of pure TB is 2.48, the titration starts with an already deprotonated molecule, and the second one is harder to measure, due to the interactions between PDADMAC and TB. In a study by Pang et al., they have investigated the possible mechanism of the generation and representation of the nonlinear interactions within the analyzed system and concluded that these variables can influence the interactions and structure disorder of the systems, depending on the environmental temperature and externally applied fields on the motions [61].

### 3.4. Responsivity Evaluations

**Figure 8:**
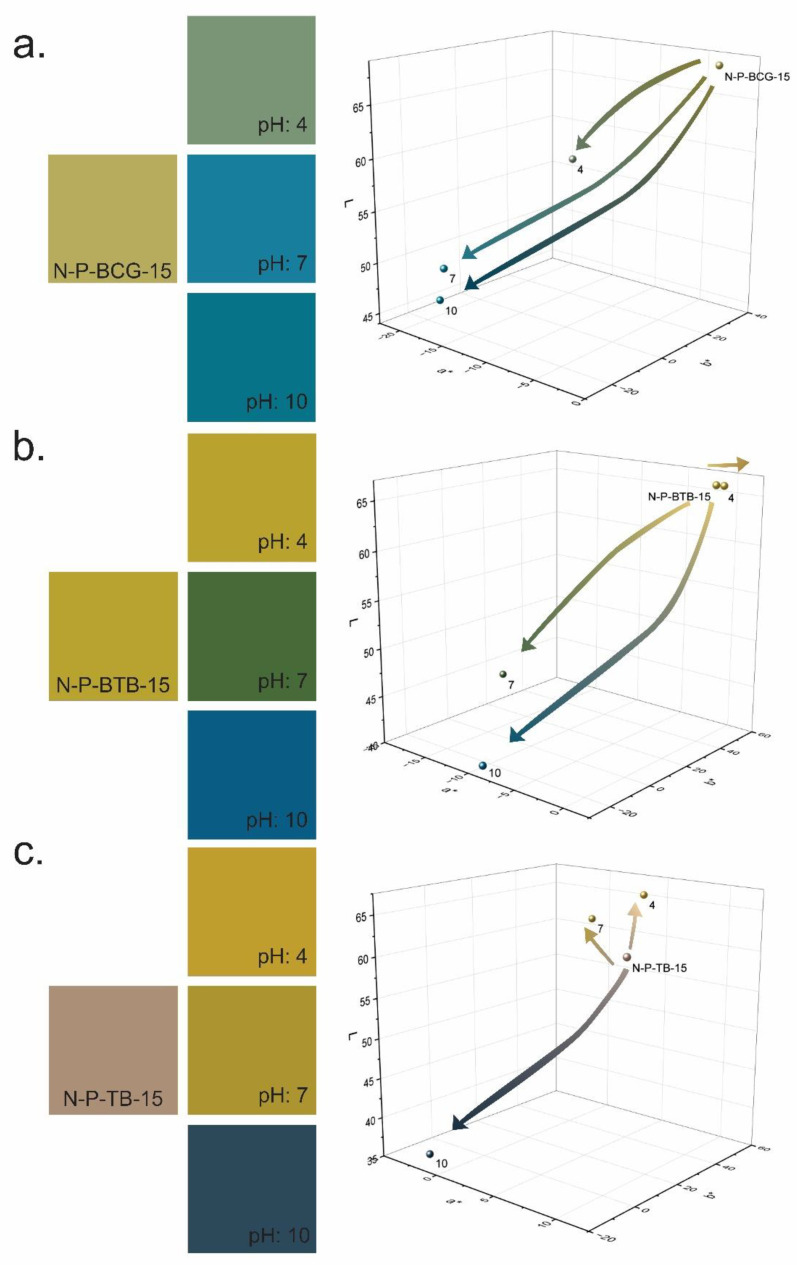
Color change of hybrid nanofibers, before and after the exposition of different pH buffers, and CIE L*a*b* results of the nanofibers exposed to different pH buffer solutions, i.e., (**a**) N-P-BCG-15, (**b**) N-P-BTB-15, and (**c**) N-P-TB-15.

To evaluate the responsivity of the dyes embedded in the nanofibers, a responsivity test was performed on electrospun nanofibrous mats. The nanofibers were exposed to buffer solutions with the selected pH values (4, 7, and 10). Figure 8 shows the nanofibers’ color change before and after they were exposed to different buffer solutions, as described in the methods section. The hybrid nanofibers responded to the exposure, according to their characteristics that are provided in Figure 1. The N-P-BCG-15 and N-P-BTB-15 samples initially showed a green/yellow color, which changed to blue, since both dyes have similar color and pH ranges, while it can be easily seen that N-P-TB-15 showed a more drastic color change. The pink/beige color became an aquamarine purple with pH 10 buffer solution exposure.

The colors of the samples were also evaluated in terms of the CIE L*a*b* color system, where L*, a*, and b* were the coordinates of the color in the mathematical combination of a Cartesian and cylindrical coordinate system, based on the theory that was explained in the previous chapter. The CIE L*a*b* color space model was employed in this study for color evaluation, since it presents a good simulation of the human vision and is independent of the device used, as well as the nature of creation [62]. As can be seen from Figure 8a, after N-P-BCG-15 is immersed in an acidic buffer (pH = 4), the a* and b* values changed from −2.90 and 35.03 to −12.27 and 11.16, respectively, thus indicating a color change towards green. When immersed in neutral pH buffer, N-P-BCG-15 changes color from green/yellow to pale blue. In this case, the a* and b* values changed to −17.28 and −21.73, respectively. Additionally, the N-P-BCG-15 changed color into blue when immersed into pH 10 buffer solution, while the a* and b* values changed into −19.17 and −17.78, respectively. Figure 1 also proves that the color changes in the nanofibers were similar to the BCG dye color range. Similarly, in Figure 8b, N-P-BTB-15 also shows BTB characteristics when changing color. N-P-BTB-15 changes color from yellow to a darker tone of yellow when immersed in pH 4 buffer solution, which was also determined by the a* and b* values, which changed from −0.59 and 50.63 to 0.24 and 50.26, respectively. Furthermore, there is a clear distinction between the green and blue range for N-P-BTB-15. When immersed in pH 7 buffer solution, a green color occurs, and the a* and b* values change into −17.42 and 18.16, respectively. When N-P-BTB-15 is immersed in pH 10 buffer solution, a blue color is observed, and the a* and b* values change into −9.85 and −23.86, respectively. Lastly, as seen from Figure 8c, when N-P-TB-15 is immersed in a pH 4 buffer solution, the pink-beige color of the nanofibers shifted into yellow, and the a* and b* values changed from 9.23 and 14.20 to 4.67 and 49.64, respectively. With pH 7 buffer solution, the nanofibers’ colors changed into yellow-green, and the a* and b* values changed into 0.90 and 45.91, respectively. Finally, an aquamarine-blue color was observed when N-P-TB-15 was immersed in a pH 10 buffer solution, and the a* and b* values changed into −2.50 and −11.68, respectively. All the results correlated with the pH color relation that the dyes have individually. Additionally, when observed with naked eye, N-P-BTB-15 shows a more distinct color change. While the change in N-P-TB-15 was not completely visible to the naked eye, the initial color of the sample immediately changed when exposed to a different pH value. Therefore, the desired responsivity was achieved.

### 3.5. Leaching Studies

As already pointed out, one of the main problems in integrating halochromic dyes into polymeric matrices is the management of leaching. The optical characteristics of the indicator dyes are very sensitive to their environment and depend on the way they are incorporated into the polymeric matrices [63,64,65]. Van der Schueren et al. showed that the use of a polymeric complexing agent (PDADMAC) in the PA6 polymer matrix significantly reduced the leaching of some pH-sensitive dye molecules, when cooperating with a PA6 polymer, since the mobility of the dye–polymer complex was lowered [15,22]. Therefore, to achieve the minimum leaching, PDADMAC was introduced to the polymeric matrix. 

**Figure 9:**
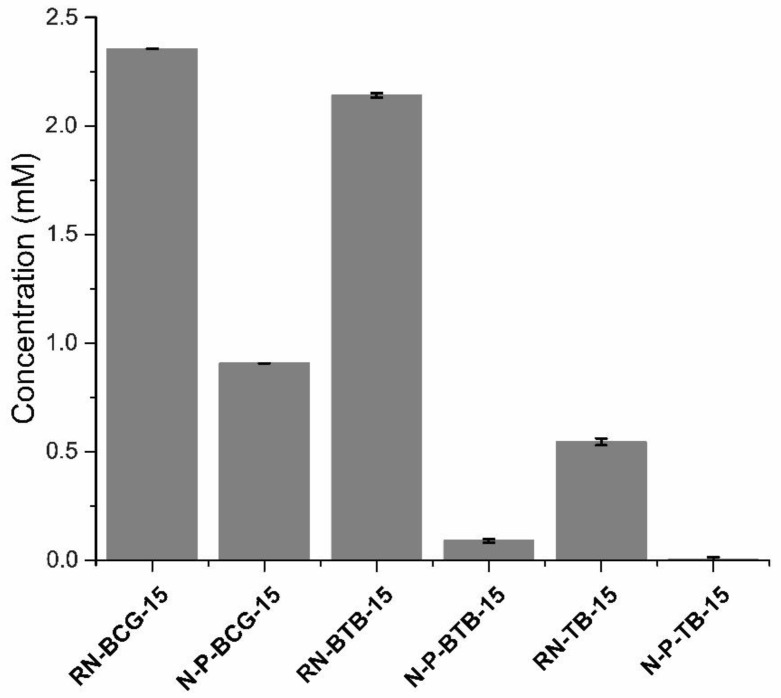
Leaching mechanism of the hybrid nanofibers and their respective references.

To investigate the characteristics of the leaching, firstly, the pure dye solutions were analyzed. The dyes were dissolved in 1× PBS and scanned using UV/VIS spectrophotometer, from 200 to 700 nm, in order to identify their maximum absorption wavelengths. The 618, 430, and 435 nm wavelengths were determined as the unique signals for the dyes BCG, BTB, and TB, respectively, and they were used in the UV/VIS analysis. The nanofibers were peeled off and weighed (0.003 g) before they were separately immersed in 5 mL separate 1× PBS solutions. A UV/VIS measurement, at a specific dye absorption maxima wavelength, was performed at the beginning of the immersion and after 24 h of nanofiber immersion. A comparison between the nanofibers, with or without the addition of the complexing agent PDADMAC, were achieved, in terms of the 24 h leaching test. The results presented in Figure 9 illustrate that PDADMAC, did affect the leaching ability of fabricated nanofibers. In the case of BCG dye, the leaching was reduced by 61%, compared to the reference nanofibers, without the addition of complexing agent. This can be caused by the electrostatic interaction between the CA and dyes, as well as the higher Br content of BCG [45]. In the case of the BTB and BTB dyes, the leaching process was significantly reduced. BTB showed a decrease of 95%, and TB showed a decrease of 99%. 

## 4. Conclusions

The present study focused on the development of polysaccharide-based nanofibrous mats with sensing ability, which can find the applications in several fields, especially biomedicine. Halochromic dyes are often used as a sensing agent in smart, responsive materials, showing a wide range of color change. Due to the insufficient integration of halochromic dyes in the polymer matrix, the leaching problem is still a pronounced issue. In our study, we overcame this problem by introducing a complexing agent, PDADMAC, thus showing a significant reduction in dye leaching. The leaching of the dye was prevented up to 99% by the addition of the complexing agent, while maintaining the color changing ability of the halochromic dyes. The study also shows that the chemical structure of the dye has a great influence on the leaching mechanism. The introduction of PDADMAC does not disrupt the nanofiber formation; in fact, the addition of PDADMAC improved the spinning ability, as well as homogeneity, of the nanofiber’s morphology.

## Figures and Tables

**Table 1:** Abbreviations and descriptions of prepared hybrid spinning solutions.

Sample Code	CA Concentration (wt%)	PDADMAC (wt%)	BCG (wt%)	BTB (wt%)	TB (wt%)
R-BCG-9	9	-	0.3	-	-
R-BTB-9	9	-	-	0.3	-
R-TB-9	9	-	-	-	0.3
R-BCG-11	11	-	0.3	-	-
R-BTB-11	11	-	-	0.3	-
R-TB-11	11	-	-	-	0.3
R-BCG-13	13	-	0.3	-	-
R-BTB-13	13	-	-	0.3	-
R-TB-13	13	-	-	-	0.3
R-BCG-15	15	-	0.3	-	-
R-BTB-15	15	-	-	0.3	-
R-TB-15	15	-	-	-	0.3
H-P-BCG-9	9	4	0.3	-	-
H-P-BTB-9	9	4	-	0.3	-
H-P-TB-9	9	4	-	-	0.3
H-P-BCG-11	11	4	0.3	-	-
H-P-BTB-11	11	4	-	0.3	-
H-P-TB-11	11	4	-	-	0.3
H-P-BCG-13	13	4	0.3	-	-
H-P-BTB-13	13	4	-	0.3	-
H-P-TB.13	13	4	-	-	0.3
H-P-BCG-15	15	4	0.3	-	-
H-P-BTB-15	15	4	-	0.3	-
H-P-TB-15	15	4	-	-	0.3

**Table 2:** Abbreviations and descriptions of electrospun nanofibers formed from their respective hybrid solutions.

Sample Code	CA Concentration (wt%)	PDADMAC (wt%)	BCG (wt%)	BTB (wt%)	TB (wt%)
RN-BCG-9	9	-	0.3	-	-
RN-BTB-9	9	-	-	0.3	-
RN-TB-9	9	-	-	-	0.3
RN-BCG-11	11	-	0.3	-	-
RN-BTB-11	11	-	-	0.3	-
RN-TB-11	11	-	-	-	0.3
RN-BCG-13	13	-	0.3	-	-
RN-BTB-13	13	-	-	0.3	-
RN-TB-13	13	-	-	-	0.3
RN-BCG-15	15	-	0.3	-	-
RN-BTB-15	15	-	-	0.3	-
RN-TB-15	15	-	-	-	0.3
N-P-BCG-9	9	4	0.3	-	-
N-P-BTB-9	9	4	-	0.3	-
N-P-TB-9	9	4	-	-	0.3
N-P-BCG-11	11	4	0.3	-	-
N-P-BTB-11	11	4	-	0.3	-
N-P-TB-11	11	4	-	-	0.3
N-P-BCG-13	13	4	0.3	-	-
N-P-BTB-13	13	4	-	0.3	-
N-P-BTB.13	13	4	-	-	0.3
N-P-BCG-15	15	4	0.3	-	-
N-P-BTB-15	15	4	-	0.3	-
N-P-TB-15	15	4	-	-	0.3

**Table 3:** Conductivity values of hybrid solutions.

Reference Sample	Conductivity (µS.cm)	Sample	Conductivity (µS.cm)
R-BCG-15	73.16 ± 0.3	H-P-BCG-15	400.40 ± 0.1
R-BTB-15	84.38 ± 0.2	H-P-BTB-15	347.40 ± 0.3
R-TB-15	38.29 ± 0.5	H-P-TB-15	304.70 ± 0.7
R-BCG-13	114.70 ± 0.1	H-P-BCG-13	448.70 ± 0.6
R-BTB-13	442.00 ± 0.7	H-P-BTB-13	711.00 ± 1.1
R-TB-13	138.25 ± 0.2	H-P-TB-13	449.60 ± 0.2
R-BCG-11	268.60 ± 0.6	H-P-BCG-11	598.80 ± 0.1
R-BTB-11	426.90 ± 0.5	H-P-BTB-11	626.90 ± 0.4
R-TB-11	88.70 ± 0.1	H-P-TB-11	352.10 ± 0.5
R-BCG-9	210.90 ± 0.8	H-P-BCG-9	343.30 ± 0.3
R-BTB-9	357.90 ± 1.2	H-P-BTB-9	481.80 ± 1.0
R-TB-9	92.40 ± 0.8	H-P-TB-9	319.10 ± 0.3

## Data Availability

Not applicable.
